# Review: How Can Intelligent Robots and Smart Mechatronic Modules Facilitate Remote Assessment, Assistance, and Rehabilitation for Isolated Adults With Neuro-Musculoskeletal Conditions?

**DOI:** 10.3389/frobt.2021.610529

**Published:** 2021-04-12

**Authors:** S. Farokh Atashzar, Jay Carriere, Mahdi Tavakoli

**Affiliations:** ^1^ Department of Electrical and Computer Engineering, Department of Mechanical and Aerospace Engineering, New York University, New York, NY, United States; ^2^ Department of Electrical and Computer Engineering, University of Alberta, Edmonton, AB, Canada

**Keywords:** COVID19, Medical Robotics, neuro-musculoskeletal disorders, telerehabilitation, smart digital health

## Abstract

Worldwide, at the time this article was written, there are over 127 million cases of patients with a confirmed link to COVID-19 and about 2.78 million deaths reported. With limited access to vaccine or strong antiviral treatment for the novel coronavirus, actions in terms of prevention and containment of the virus transmission rely mostly on social distancing among susceptible and high-risk populations. Aside from the direct challenges posed by the novel coronavirus pandemic, there are serious and growing secondary consequences caused by the physical distancing and isolation guidelines, among vulnerable populations. Moreover, the healthcare system’s resources and capacity have been focused on addressing the COVID-19 pandemic, causing less urgent care, such as physical neurorehabilitation and assessment, to be paused, canceled, or delayed. Overall, this has left elderly adults, in particular those with neuromusculoskeletal (NMSK) conditions, without the required service support. However, in many cases, such as stroke, the available time window of recovery through rehabilitation is limited since neural plasticity decays quickly with time. Given that future waves of the outbreak are expected in the coming months worldwide, it is important to discuss the possibility of using available technologies to address this issue, as societies have a duty to protect the most vulnerable populations. In this perspective review article, we argue that intelligent robotics and wearable technologies can help with remote delivery of assessment, assistance, and rehabilitation services while physical distancing and isolation measures are in place to curtail the spread of the virus. By supporting patients and medical professionals during this pandemic, robots, and smart digital mechatronic systems can reduce the non-COVID-19 burden on healthcare systems. Digital health and cloud telehealth solutions that can complement remote delivery of assessment and physical rehabilitation services will be the subject of discussion in this article due to their potential in enabling more effective and safer NMSDK rehabilitation, assistance, and assessment service delivery. This article will hopefully lead to an interdisciplinary dialogue between the medical and engineering sectors, stake holders, and policy makers for a better delivery of care for those with NMSK conditions during a global health crisis including future pandemics.

## 1 Introduction

Worldwide, over 127 million cases of patients with a confirmed link to COVID-19 and about 2.78 million deaths have been reported at the time this article was written (Johns Hopkins University. (2020)). With limited access to vaccine or strong antiviral treatment for the novel coronavirus, actions in terms of prevention and containment of the virus transmission rely mostly on social distancing among susceptible and high-risk populations (Block et al. (2020); Lewnard and Lo (2020); World Health Organization. (2020)). Also, mitigation strategies among suspicious and positively tested populations again rely on isolation measures, with the exception of those who are sufficiently ill to be hospitalized (Jawaid (2020); Tripathy (2020)). This review paper focuses on elderly adults with acute or chronic neuro-musculoskeletal disorders and disabilities.

Aside from the direct challenges posed by the novel coronavirus pandemic, there are serious and growing secondary consequences (explained below) caused by physical distancing, isolation guidelines, and by focusing the healthcare resources almost only on COVID-19 (Bartolo et al. (2020)). Related to the mentioned consequences, it should be noted that the healthcare system’s resources and capacity have been focused on addressing the COVID-19 pandemic, causing less urgent care, (e.g. physical neurorehabilitation and assessment) to be paused, canceled, or delayed, resulting in non-COVID health-related concerns for patients suffering from other conditions, such as post-stroke disabilities (for which intense and immediate rehabilitation is needed). However, In many jurisdictions, in-person visits to rehabilitation clinics were prohibited with the exception of serious emergency cases; thus, at best, non-emergency assessment and rehabilitation were transitioned to remote delivery via verbal or visual teleconferencing (please see Caso and Federico (2020); Ferini-Strambi and Salsone (2020); Leocani et al. (2020); Ng et al. (2020); Seiffert et al. (2020); Srivastav and Samuel (2020); Venketasubramanian (2020)). As a result, this has left the elderly and adults with acute and chronic conditions, in particular those in need of receiving neuromusculoskeletal rehabilitation services, without the required support resulting in serious delays for therapeutic and rehabilitation services (Schirmer et al. (2020)). This has also resulted in delays between the appearance of symptoms of a non-COVID life-threatening condition (such as stroke or heart attack) and when patients seek urgent care (Lange et al. (2020); Kansagra et al. (2020)). Unfortunately, in many cases, such as stroke, fast initiation of treatment and prompt followup rehabilitation services are critical, since 1) late initiation of therapy can result in vaster damage, and 2) neural plasticity after stroke decays very quickly with time. In addition, in many cases, care for non-life-threatening chronic disabilities and illnesses has been deferred to the future, creating a backlog that will take years to clear. All of these put an excessive amount of pressure on the infrastructure of society including healthcare systems in various domains which are now serving for the fight against the virus among the society.

Given that multiple waves of the outbreak are expected (Stefana et al. (2020); Xu and Li. (2020)) in the coming months worldwide, it is important to address this issue as societies have a duty to protect the most vulnerable populations. The actions which are being taken during this process will be imperative to boost up our healthcare system and make it prepared not only for future waves of this pandemic but also for future pandemics. The COVID-19 pandemic has shown that our current healthcare system and model of healthcare delivery are far more unprepared (King. (2020)) than anticipated and require rethinking and substantial future preparation in order to provide continuity of care throughout the second and third waves of COVID-19 and for potential future pandemics.

In this article, we provide a detailed and targeted analysis of the literature based on which we argue that intelligent robotics and smart wearable technologies can help with extended, accessible, and remote delivery of assessment and rehabilitation services while physical distancing and isolation measures are in place to curtail the spread of the virus. We will also discuss that through supporting patients and medical professionals during this pandemic, robots, and smart mechatronic systems (such as telerobotic rehabilitation platforms), which have been designed in the literature and can be exploited here, have the potential to reduce the non-COVID-19 burden on healthcare systems so that the hospitalization and treatment of COVID-19 patients can remain the top priority.

This article conducts a literature survey supporting the use of robotics technologies and AI for enhancing the quality of care delivery specially for patients with NMSK conditions. This is motivated by the fact that, in times of deep health crises such as during the novel coronavirus pandemic, medical robotic and smart wearable systems can play a positive role by assisting the healthcare system and safeguarding public health in various ways. Within this review we define smart wearable systems as wearable IoT type devices, (e.g. a FitBit) which contain various sensors and can provide feedback (through visual or other means) to the patient. We will discuss exoskeletons separately, given their utility for rehabilitation and assistance. Another robotic modality we will discuss are telerobots, which can enable closed-loop, autonomous, and semi-autonomous kinesthetic interaction between an in-home patient and in-clinic therapies for rehabilitation exercises of stroke patients (Atashzar et al. (2016a); Shahbazi et al. (2016); Atashzar et al. (2018); Hooshiar et al. (2019); Panesar et al. (2019); Fong et al. (2020a); Fong et al. (2020b); Sharifi et al. (2020)). In addition, robots and telerobots can be used to help in preventing the spread of COVID-19 by making it possible for frontline healthcare workers to screen, triage, evaluate, monitor, and even treat patients from a safe distance (please see Tavakoli et al. (2020) for a high-level review of how robotics can aid the healthcare workers, and society). In this regard, digital health and telehealth solutions that integrate assessment and physical rehabilitation of people with chronic NMSK conditions are the focus of this review article and will be the subject of discussion below due to their potential in enabling more effective and safer NMSK rehabilitation and assessment service delivery. We will present examples of robotic systems that aid and complement remote delivery of assessment and physical rehabilitation services for adults with chronic conditions.

It should be highlighted that this paper is written based on the lessons we learned from COVID-19, in particular the deficiency of remote rehabilitation and assessment for patients considering a wide demographics. COVID-19 has proven that our healthcare system is not prepared for taking such an unprecedented challenge. This paper examines not only the current activities but also the future horizon of technology and investigates how can intelligent robots and smart mechatronic modules facilitate remote assessment, assistance, and rehabilitation for isolated adults with NMSK conditions. The last sentence is indeed the title of the paper to show that we not only consider direct challenges caused by COVID-19 but also we look beyond COVID-19 to broaden the knowledge on the potentials for the existing technologies to martialize the health care of tomorrow.

In addition to discussing existing rehabilitation and assistive technologies for a more efficient delivery of care for individuals with NMSK disabilities, we also discuss where there is potential for further use of this technology to improve the quality of life among this population. This will hopefully lead to an interdisciplinary dialogue between the medical and engineering communities in addition to the end-users of these technologies, i.e., people in long-term or home care with chronic NMSK conditions. This article also attempts to open a line of conversation, supported by strong literature, between the public, stakeholders, and policymakers about the real, practical, and life-saving benefits that can be achieved in a short-term future with the use and fusion of existing robotic, telerobotic, and wearable technologies in the healthcare system.

It should be highlighted that, before the pandemic era, robotics and automation were often tagged in several analyses as a force that can eliminate jobs and damage humanity and society. This article represents a targeted and focused literature review to impress upon the fact that at this time, more than ever, we need to invest in and investigate the life-saving potentials of robotics and AI to better serve our society and reduce the burden on healthcare systems during such unprecedented situation. A science-based ethics-centered shift of culture toward more advanced use of technology to assist delivery of healthcare services (and in particular those related to NMSK conditions) requires increasing the awareness about the features of existing technologies, besides, dialogue, and collaboration. This perspective review article aims to be one step in that direction.

## 2 Population Aging Before COVID-19: An Underlying Compounded Problem

Based on official numbers and statistics, the population of senior adults worldwide over the age of 60 is expected to more than double by 2050. It is anticipated that by 2047, the number of senior adults will exceed the number of children. This trend is expected to continue due to increased life expectancy and reduced fertility rates. An aging society can become a global public health challenge in the near future and have significant social and economic effects on healthcare systems worldwide (Christensen et al. (2009); Chatterji et al. (2015); Suzman et al. (2015); World Health Organization (2015)). The rapid aging of societies worldwide is likely to increase the incidence rate of age-related neuromuscular and sensorimotor degeneration and corresponding disabilities. These age-related neuro-muscular disabilities are caused by various factors such as normal degeneration, stroke, and musculoskeletal conditions, resulting in sensorimotor dysfunction (Degardin et al. (2011)), impaired mobility (Wesselhoff et al. (2018)), and long-lasting motor disabilities (Alawieh et al. (2018)), directly affecting the quality of life of senior adults (Almkvist Muren et al. (2008)). In addition to the deleterious effect on the quality of life, these disabilities can reduce life expectancy, increase the risk of injuries (particularly fall-related injuries), and result in further cognitive and sensorimotor deterioration.

Stroke is the leading cause of significant age-related neuromuscular and sensorimotor impairment (Mukherjee and Patil (2011); Prince et al. (2015); Mozaffarian et al. (2015)) and causes excessive pressure on healthcare systems. This has been a major concern even before the substantial extra pressure due to the pandemic. Many stroke survivors experience permanent or long-lasting motor disabilities and often require labor-intensive sensorimotor rehabilitation therapies and progress monitoring during the golden time of recovery, the acute post-stroke phase, and an extended period of time afterward (Dimyan and Cohen (2011); Teasell and Hussein (2016)). The need to rapidly begin treatment after a stroke and the extended duration of treatment for stroke patients (Arias and Smith (2007); Cumming et al. (2008); Cumming et al. (2011); Yen et al. (2020)), places a significant burden on the healthcare system. The likely outcome is that, with a healthcare system that is already under-resourced, many patients suffering from a significant functional deficit would not receive sufficient rehabilitation and progress monitoring services during the pandemic, when the healthcare system is extensively loaded with managing (and preparing for) COVID-19 patients.

For a broad range of NMSK disabilities, it has been shown that rehabilitation technologies, including multimodal biofeedback, functional electrical stimulation therapy, and intelligent robotic rehabilitation systems can significantly help patients in regaining some of the lost sensorimotor functionalities (please see Takeda et al. (2017); Atashzar et al. (2019); Yang. et al. (2019b) and references therein). These rehabilitation technologies have been seen as an adjunct to traditional rehabilitation therapies, and may potentially replace traditional therapies for accelerating neural plasticity and regaining lost sensorimotor function, which results in increasing functional capacity, quality of life, and ultimately patient independence. The concern of societal aging and age-related NMSK disorders is more pronounced due to the current pandemic. Most of the patients in need of urgent and long-term NMSK rehabilitation services are senior adults who are in the vulnerable category considering the demographics related to COVID19. The question is, “how can we deliver rehabilitation services to this population during, and after COVID19 pandemic?” This question has raised in a serious international conversations on how to deliver acute stroke rehabilitation during the pandemic (please see the following citations and references therein Lyden. (2020); Rudilosso et al. (2020); Smith et al. (2020); Wang et al. (2020)). The problem is that a long delay can result in losing major motor functionality, which would not happen if rehabilitation was delivered in a timely manner, minimizing permanent damages. A systematic literature-based investigation on this question to find alternative solutions can highlight the use of Robotics and AI technologies for rehabilitation, which is the focus of this article and can help with addressing the excessive pressure on the healthcare systems resulting in interruption of neurorehabilitation for patients in need.

## 3 Categories of Robotic Systems for Boosting Care Delivery

**FIGURE 1:**
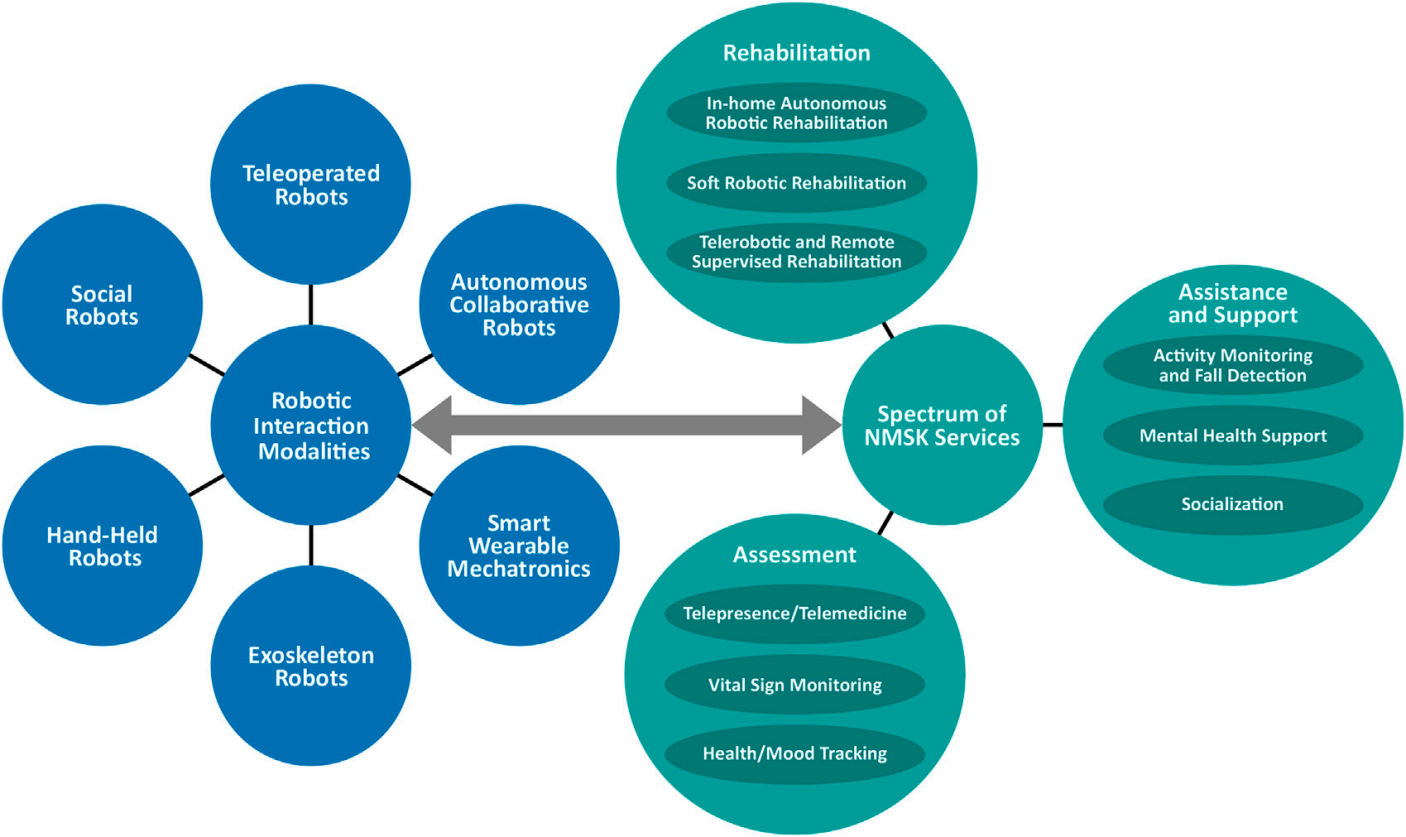
Categories of robotic interaction and example remote rehabilitation, assessment, assistance, and support tasks for adults with neuro-musculoskeletal conditions.

Figure 1 demonstrates the overall design of the paper and shows how various modalities of robotics can be used for three main modalities of the healthcare spectrum (rehabilitation, assessment, and assistance) needed for patients with NMSK disabilities during and after a pandemics. In Figure 1, we categorize various robotic systems and various modalities of care. Some robots can be used for multiple modalities of care. For example, an exoskeleton can be used to retrain a post-stroke patient when the patient performs a wide range of robotics-enabled treadmill based task in a virtual reality environment so that gradually the patient’s nervous system can be retrained and the patient can walk better out of the robot. For this, the physical, intensity, and temporal characteristics of robotic therapy should be designed in a way that maximizes the engagement of the patient and stimulation f the nervous systems. An example of this technology is Locomat from Hocoma (Switzerland). In addition, the exoskeleton can be used as an advanced wheelchair in the format of an assistive device, the primary function of which is to help the patient to perform the activities of daily living with the use of the robot without being too concerned about retraining the brain. In this regard, the robot should be able to detect the intention of the patient and help to perform the task for the patient. Another example is social robotic systems for kids with cerebral palsy, which has shown potential for helping this population to better engage in sensorimotor learning activities over time of aging as a rehabilitative device. Also, social robots are used for elderlies to assist them in managing isolation in long-term care facilities (as an assistive device). Figure 1 shows the overall concept of the paper when we classify the modalities of robotic systems and modalities of care services, emphasizing that robotic systems can be used in a variety of health care application, while some format of robotic systems can have multiple health care application and some may have one or few applications. In this paper, based on the concept shown in Figure 1, we will discuss different robotic modalities which have been used for a wide range of spectrum of care for patients with NMSK conditions. In the current section, categories of robotic systems are introduced for boosting the care delivery, while Sections 4, 5 and 6 will provide relevant discussions about the use of robots for addressing the mentioned spectrum during and after COVID-19 with the focus on patients living with NMSK.

In the literature, a wide range of robotic systems and wearable technologies have been introduced to help people with NMSK conditions. In order to establish an efficient discussion about the existing technologies and how they can be adapted to help with the current pandemic situation, it is advantageous to discuss a number of definitions and ways to classify such technologies. Categories can be defined according to either 1) *mechanical structure* or 2) *modality of human-robot interaction (HRI)*. The former explained the mechanical characteristics of the robots regardless of how it interacts with humans, while the latter focuses on how these systems physically and intelligently interact with humans to deliver the needed care. In this article, the modality of interaction is considered to be the primary distinguishing factor between various robotic and wearable systems. The resulting categories can be defined as *Telerobots, Autonomous Collaborative Robots, Exoskeleton Robots, Smart Wearable Mechatronic Systems, Hand-held Robots, and Social Robots*. The proposed categorization (which takes into account the interaction, intelligence, and control) helps to lead the discussion on how particular styles of robotic systems can assist with the three core modalities of the spectrum of healthcare for NMSK patients, during the COVID19 pandemic, namely, *assessment, rehabilitation, assistance*.

The intersections between various human-robot interaction modalities and the spectrum of healthcare delivery are shown in Figure 1. In this article, we provide literature-based discussion and our perspective on how HRI categorizations can help the healthcare system during and after the COVID-19 pandemic. In this section, we also offer some examples corresponding to a subset of possible robotic solutions existing at these intersections. The hope is that this review of existing technologies starts an in-depth discussion and inspires others to quickly find new and innovative solutions using existing systems in the literature that can be applied across the healthcare spectrum and using all possible modalities of human-robot interaction in the era of the current crisis and to prepare for future waves and future pandemics. To help the reader we have created Table 1, which is a summary of the following section. Table 2 contains selected references from the literature to show which type of robotic systems are commonly applied to the three healthcare tasks covered in this review, (i.e. Rehabilitation, Assessment, and Assistance/Support).

**TABLE 1:** Summary of advantages and limitations of robotic interaction modalities.

		Advantages	Limitations
Robotic interaction modalities	Teleoperated robots	Remote operation; sensory augmentation through data fusion; motor augmentation; bypassing the barrier of distance; computerized interaction to log the performance metrics of both users at the two terminals	Minimum to no autonomy; concerns regarding transparency of reflected force field; susceptibility of system stability to network time delay and the variation in the delays which may challenge safety; relatively high cost due to the need for two robots; synchronization challenges
	Autonomous collaborative robots	High level of autonomy; need for minimum-to-no intervention from human; allowing for higher level of distancing; possibility of infinite work space (for mobile systems); can be integrated with existing mechanical and mechanic systems such as wheelchairs; securing a high level of sensor-based situational awareness; minimizing possible human error (depending on the context) relying on the past data and cloud computation	Totally removing the human domain knowledge from the loop which can raise safety risks for unseen situations and under unstructured conditions; susceptibility to sensor failure; susceptibility to biases in the data sets based on which a behavior is trained; need for extra and redundant sensors with high speed which can increase the cost and accessibility
	Exoskeleton robots	Joint-space operation for augmenting the natural motor ability of users; augmenting the mechanical power of the wearer and enhancing the safety; ability to serve as both assistive and rehabilitative system; reducing the mechanical load on the joints, skeleton, and muscles of the users (such as workers) supporting a high level of musculoskeletal health	Need for high power; increasing the weight and battery size; major concerns of safety due to the several point of physical contacts with the user and due to the secured contacts with the user; a high level of safety risk in the case of sensor failure; high cost; low accessibility; low level of compatibility (the current state) with various unstructured environments
	Smart wearable mechatronic systems	Ability to be worn and measure body signals; ability to provide biofeedback through due to close skin contact; augmenting sensory awareness (haptics and proprioception); ability to measure body motion for monitoring and rehabilitation in the context of supervised or unsupervised telemedicine; ability to contact tracing and localization for navigation and for medical purposes; ability to communicate with cloud over internet (in the context of IoT)	Low battery life and need for recharge in case of high functionality due to limited space; possibility of errors in measurement due to the small and variable surface contact (such as due to hair blockage or sweating) resulting in false-positive and false-negative alarms/reports; susceptibility to hacking and attacks when communicating biological signals and location information over cloud; limited actuation ability due to the limited power and size
	Hand-held robots	Being light-weight while powered; providing active assistance to delicate manual tasks; application in helping people with hand tremor as an eating assistive device for higher independence	Limitation complex mechatronic design of sensors and actuators due to the small size and limited acceptable weight; relatively high cost; limited degrees of freedom; limited number of tasks which can benefit
	Social robots	Interact socially with humans including patients with cognitive disorders or those in isolation; providing sense of social engagements; supporting education and development for kids with autism; possibility of multiple recording during social engagement (including mood, stress and vital signs)	Limited actuation and degrees of freedom needed for a natural social interaction; challenges to adapt to complex cognitive-related factors affecting social interaction; requirement for a very high level of intelligence to promote social engagement

**TABLE 2:** Categorization of selected articles from the literature.

		Healthcare services		
		Rehabilitation	Assistance and support	Assessment
Robotic systems	Teleoperated robots	Atashzar et al. (2016a); Shahbazi et al. (2016); Atashzar et al. (2018); Panesar et al. (2019), Fong et al. (2020b); Sharifi et al. (2020)	Pernalete et al. (2002); Pernalete et al. (2003); Atashzar et al. (2017a); Reis et al. (2018); Hooshiar et al. (2019); Mehrdad et al. (2021)	Brennan et al. (2009); Fong et al. (2020a); Kim et al. (2020)
	Autonomous collaborative robots	Krebs et al. (1998); Krebs and Hogan. (2006); Brewer et al. (2007); Blank et al. (2014); Maciejasz et al. (2014); Pehlivan et al. (2016); Díaz et al. (2018); Atashzar et al. (2019); BionikLabs. (2020); Nicholson-Smith et al. (2020)	Chow and Xu. (2006); Parikh et al. (2007); Leaman and La. (2017); Chen et al. (2018a); Wu et al. (2019); Azad et al. (2020)	Balasubramanian et al. (2012); Debert et al. (2012); Lambercy et al. (2012); Nordin et al. (2014); Otaka et al. (2015); Kuczynski et al. (2016); Kuczynski et al. (2017); Simbaña et al. (2019); Simmatis et al. (2019); Simmatis et al. (2020)
	Exoskeleton robots	Mao and Agrawal (2012); Proietti et al. (2016); Bernocchi et al. (2018); Rehmat et al. (2018); Bao et al. (2019); Shi et al. (2019); Hocoma. (2020)	Chen et al. (2013); Pazzaglia and Molinari (2016); Randazzo et al. (2017); Shore et al. (2018); Di Natali et al. (2019); Lyu et al. (2019); Kapsalyamov et al. (2020); Settembre et al. (2020)	Ball et al. (2007); Rocon et al. (2007); Fitle et al. (2015); Simmatis et al. (2017); Rose et al. (2018); Mochizuki et al. (2019)
	Smart wearable mechatronic systems	Bonato (2005); Polygerinos et al. (2015); Simon et al. (2015); Yang et al. (2018); Bisio et al. (2019); Kos and Umek (2019); Wei et al. (2019)	Shull and Damian (2015); Katzschmann et al. (2018); Sweeney et al. (2019); Gathmann et al. (2020); Alva et al. (2020); Seshadri et al. (2020)	Šlajpah et al. (2014); Qiu et al. (2018); Qiu et al. (2019); Carnevale et al. (2019); Cerqueira et al. (2020); Oubre et al. (2020)
	Hand-held mechatronic systems and robots	Rinne et al. (2016); Hussain et al. (2017); Mace et al. (2017)	MacLachlan et al. (2011); Pathak et al. (2012); Yang et al. (2014); Pathak et al. (2014); Sabari et al. (2019); Ripin et al. (2020)	Rinne et al. (2016); Hussain et al. (2017); Mace et al. (2017)
	Social robots	Fasola and Mataric (2012); Calderita et al. (2013); Malik et al. (2016); Céspedes et al. (2020); Martín et al. (2020)	Broekens et al. (2009); Belpaeme et al. (2018); van den Berghe et al. (2019); Scoglio et al. (2019); Armitage and Nellums (2020)	Pennisi et al. (2016); Chen et al. (2018b); Do et al. (2020)

### 3.1 Teleoperated Robots

These systems are composed of two synchronized robotic systems (often called as leader-follower robotic systems, or leader and follower robotic consoles) that communicate over a communication channel (see Avgousti et al. (2016); Niemeyer et al. (2016); Farooq et al. (2017); Evans et al. (2018); Hooshiar et al. (2019) and references therein). An extension of these technologies are multilateral telerobotic systems (see Shahbazi et al. (2018) and references therein) which have multiple robots interacting over a multiport network, realizing collaborative tasks by operators or robots or both. The communication channel can be a hard line, or satellite, or the internet. The purpose of such technology is to transfer the agency and motor control of the human operator(s) over a barrier and allow remote operation while receiving sensory awareness feedback from the remote environment(s) for the operator(s). Four main examples of barriers are distance, danger, safety, and scale. A successful example of a translational telerobotic technology in a totally different medical application, (i.e. surgery) is the da Vinci surgical robotic system.

In the context of NMSK, emerging telerobotic rehabilitation systems which recently have attracted a great deal of interest (Atashzar et al. (2016a); Shahbazi et al. (2016); Atashzar et al. (2018); Hooshiar et al. (2019); Panesar et al. (2019); Fong et al. (2020a); Fong et al. (2020b); Sharifi et al. (2020)) allow remote access of patients to kinesthetic rehabilitation and remote monitoring under telemedicine, maximizing accessibility regardless of geographical barrier and minimizing the risk associated with commuting to healthcare centers. This topic is discussed in details later in this paper (under Sections 4.3, and 4.4).

### 3.2 Autonomous Collaborative Robots

These technologies are designed particularly to physically conduct a task with the need for a high level of autonomy, and situational awareness, and in collaboration with human operators. Several examples and the literature can be found in (Ajoudani et al. (2018); Chen. et al. (2018a); Saenz et al. (2018); Haidegger. (2019); Hentout et al. (2019); Gualtieri et al. (2020)). These robots sometimes have fixed bases, sometimes have mobile bases, and sometimes they are equipped with arms. In addition, hybrid collaborative arm systems exist, having one end fixed to a mobile base, which is free to perform tasks in an environment dexterously (these are often called mobile manipulators). Mobile manipulators allow for a theoretically infinite workspace for the manipulator (see the following citations for more information about the modern application of this technology: Zhao et al. (2018); Wu et al. (2019); Balatti et al. (2020)). Such hybrid robotic systems can be used in healthcare centers for manipulating and moving materials, and even can assist with delivering physical assistance for patients, reducing physical interaction between personnel and between patients and caregivers. Autonomous collaborative robots have been used frequently in industry, and more recently in health care systems (motivated by the need to such technologies for handling COVID-19-related issues), to reduce the load of repetition and precision when collaboratively conducting tasks with humans. There are a wide range of examples, but one particular example is handling samples of COVID-19 and being part of the testing pipeline, making the whole testing chain faster and more reliable (please see Yang et al. (2020) for more details). In addition to the above, mobile platforms (typically without manipulators), including smart wheelchairs, are not fixed in a position and instead use a wheeled platform or walking mechanism to move in an environment (Chow and Xu (2006); Parikh et al. (2007); Leaman and La. (2017)). This technology can be used for various applications, including 1) mobility of patients with physical NMSK disability and those with reduced cognitive strength caused by COVID-19, reducing the need for physical assistance by human, and maximizing patients’ independence; 2) as an inherent part of telemedicine which can be used for delivering care remotely and checking vital signals in isolated centers (such as nursing homes); and 3) interaction between isolated patients and their families and personnel of the facility.

### 3.3 Exoskeleton Robots

These robots are external actuated mechanisms worn by humans for motor augmentation, strengthening the users’ capabilities, or to rehabilitate a human’s lost abilities and function (Gopura et al. (2016); Proietti et al. (2016); Young and Ferris (2016); Hill et al. (2017); Rehmat et al. (2018); Di Natali et al. (2019); Settembre et al. (2020)). Using such technical aspects of rehabilitation and mobility can be realized with minimum human-based intervention. Exoskeletons have been used in industries to reduce the mechanical load on workers. With the same functionality, they have been proposed to be used for assisting patients with extreme mobility problems, and in this regard, they have been often seen as the next revolutionary generation of wheelchairs (Pazzaglia and Molinari. (2016); Hill et al. (2017)). They have been designed in various formats, including upper-limb and lower limb, and combined. Using exoskeleton patients with NMSK disabilities can be rehabilitated during walking and mobility exercises while finely tuning the characteristics of exercise (including the speed, step length, joint trajectories, posture). This will significantly reduce the need to have multiple therapists closely interacting with a patient to deliver the mobility exercises.

### 3.4 Smart Wearable Mechatronics

These technologies are human-worn devices that measure body signals and display information to the user through biofeedback to support, assist, or augment the capabilities of the user. Smart wearables can also provide haptic-, vibro-, and electro-feedback stimulation to users (see the following citations for examples and more details: Polygerinos et al. (2015); Chen et al. (2017); Maisto et al. (2017); Yang. et al. (2019a); Alva et al. (2020); Cerqueira et al. (2020); Gathmann et al. (2020)). These technologies have been used to enhance the sensory capability of patients with NMSK disabilities (such as Simon et al. (2015); Lopes and Baudisch (2017); Bisio et al. (2019); Alva et al. (2020); Gathmann et al. (2020)). These technologies have also been categorized under the umbrella of the Internet of Medical Things (IoMT) (Bisio et al. (2019))) and smart environments. Related to COVID-19, recently, researchers are utilizing wearable technologies for following the time-series of symptoms of patients, especially those with NMSK disabilities which may degrade the ability to monitor the symptoms through traditional means, and evaluate the evolution and dynamics in bio-markers. These wearable sensor technologies have the potential to provide early diagnosis of those who may be in a sensitive age range or with underlying conditions; also for monitoring of those who have shown some symptoms but not serious enough to be hospitalized. With the use of artificial intelligence, the collected data can be processed on the cloud, and any health anomaly can be detected using computational models (see examples: Saglia et al. (2019); Ding et al. (2020); Seshadri et al. (2020); Weizman et al. (2020); Tripathy et al. (2020)). As mentioned, these technologies can be equipped with the tactile actuator to provide sensory feedback for the user, for example when they move their hand close to their face (D’Aurizio et al. (2020)), or when they do not follow guidelines for washing the hands for a long enough duration; providing an additional layer of situational awareness. These technologies can also be used to track the spread of the virus by tracking the mobility of those with comorbidities. In this regard, recently, there have been several conversations about data security and privacy of the users, which are all ongoing topics at the moment, to make sure that these technologies follow the ethical guidelines and privacy of the users (Arias et al. (2015); He et al. (2018); Tseng et al. (2019); Stoyanova et al. (2020)).

### 3.5 Hand-Held Robots

This is a relatively small category of assistive robotic systems. These technologies are light-weight powered robotic systems designed to be held in a user’s hand and typically assist with performing tasks. Initial uses of hand-held robotics were in surgery to help a surgeon stabilize physiological hand tremors when performing delicate surgical operations, such as retinal surgery (MacLachlan et al. (2011); Becker et al. (2013); Yang et al. (2014)). Recently, the same concept has been utilized to assist patients with NMSK disabilities, in particular, assisting users with severe NMSK disabilities when eating. This reduces the need for interaction with nurses and other helpers (family members), enhancing the independence and quality of life of users. An example of such a robot is a smart-spoon, which counteracts hand tremors in those with Parkinson’s disease to allow them to eat more easily with more confidence and without the need for someone to feed them (Pathak et al. (2014); Stamford et al. (2015); Sabari et al. (2019)). Such technology not only helps with a patient’s self-confidence and mental state but also, during the COVID-19 pandemic, it will reduce the need to have close and long physical interaction with nurses and helpers for feeding (as one example).

### 3.6 Social Robots

These technologies are robots that interact socially with humans (Campa. (2016)) and have been used for a variety of applications that benefit from social interaction, such as for education (see Belpaeme et al. (2018) and references therein), for language learning (see van den Berghe et al. (2019) and references therein), for elderly care (see Broekens et al. (2009) and references therein), for helping people with autism (see Pennisi et al. (2016) and references therein), and depression (see Chen. et al. (2018b) and references therein). Social robots may be actuated or have speech capabilities and can measure the user’s mood, temperature, stress, and vital signs via various embedded sensors. Smart social robots have shown good potential in engaging the users in interactive social exercises. Social robotics systems have been shown to successfully benefit kids living with autism (Pennisi et al. (2016)), and elderly living with mild cognitive impairments, Alzheimer’s disease, and dementia (Valentí Soler et al. (2015); Góngora Alonso et al. (2019)). This technology can be a major benefit, especially during the COVID-19 pandemic, when the elderly are isolated due to the concerns over disease spread. Long term isolation for patients who are already having cognitive disorders may have very serious consequences, and any technology which can engage these persons in interactive social exercises, while reducing the risk of human-human contact, can be significantly beneficial.

## 4 Rehabilitation Robotics

### 4.1 Rehabilitation During the COVID-19 Pandemic and Post-COVID Era

As mentioned earlier, the COVID-19 pandemic has put high pressure on healthcare systems. Due to the inability of patients to visit rehabilitation centers, or the risk of patients when going to rehabilitation centers, the delivery of NMSK rehabilitation has been distorted. It should be noted that most patients who have experienced stroke(s) have an age greater than 65. This means that the population of stroke patients is categorized as at-high-risk, and it is critical for those patients to minimize situations that may result in human contact, in particular visits to health care systems. Concern has been raised, since the delivery of rehabilitation is a time-sensitive treatment (as mentioned in the introduction). A delay, or long pause, in treatment can result in permanent loss of major sensorimotor functionality. Recent literature strongly suggests very early mobilization and intense therapy right after stroke to secure a high degree of functional recovery, during the short golden time (right after the stroke) when brain plasticity is at its maximum (Arias and Smith (2007); Yen et al. (2020); Cumming et al. (2008); Cumming et al. (2011)). However, currently, COVID-19 is the main (if not sole) focus of healthcare systems in many countries. Thus, while there are many patients who experience a stroke during this very challenging time, access to healthcare facilities is strictly limited. Also, as mentioned in the introduction, not only has the pressure of COVID-19, and corresponding concerns about disease transfer to the elderly, resulted in delays in delivery (and consistency of delivery) of rehabilitation services, but also the fear of COVID-19 has caused delays where patients are holding off in seeking emergency care after stroke symptoms. It should also be pointed out that family members, who usually play a central role as the regular caregiver (or helper) for the post-stroke process, are usually partners of an age that also likely falls within the high-risk category for COVID-19. Thus, it would be highly risky (if not impossible) for patients and their immediate families to travel repeatedly to healthcare centers to receive frequent rehabilitation services. At the same time, it is highly risky for post-stroke patients to remain in the hospital as in-patients, due to the risk of pneumonia, which can be significant for those with suppressed immune systems. Thus, now, the question is how we can use the existing intelligent robotic and mechatronic technologies, and how we can expand and exploit them to deliver a high degree of care while maximizing patients’ safety.

### 4.2 Conventional Robotic Rehabilitation

A solution suggested in the literature, before the current COVID-19 pandemic, for reducing pressure on the healthcare system to deliver labor-intensive rehabilitation was to develop in-clinic robotic technologies that provide repetitive, multimodal, rehabilitation exercises (such as active assist robot, and exoskeletons for both upper and lower limbs). Examples of such robots are InteractiveArm (which is an upper limb end-point robotic system from BionikLabs, Toronto, Canada (BionikLabs. (2020))), ArmeoPower (which is an upper limb exoskeleton from Hocoma, Switzerland (Hocoma. (2020))). Robotic rehabilitation technologies are designed to promote multimodal stimulation of neural and muscle activities, while patients perform tasks in a virtual-reality environment. Functionality, effectiveness, and various formats of robotic rehabilitation are explained in our recent literature survey, published in (Atashzar et al. (2019)). Conventional robotic rehabilitation technologies utilize various modalities of interaction, mainly being collaborative robots (Peternel et al. (2017)) and exoskeletons (examples can be found in Proietti et al. (2016); Rehmat et al. (2018); Lv et al. (2018); Lefeber et al. (2019)). Commercial robotic rehabilitation technologies are composed of three components:

a) A sensorized robotic module which is an active medical device and can provide multi-directional and high bandwidth kinesthetic force fields (such as assistive, coordinative, and resistive forces) and vibrotactile haptic feedback, to enable the delivery of various types of rehabilitation for patients with a wide range of biomechanics, motor deficits, and levels of muscle tone, spasticity, and involuntary motions. A core design factor is to make the robots responsive to allow for rendering a highly transparent and agile interaction with the patient’s biomechanics, which is an imperative factor for an efficient rehabilitation regimen. Rehabilitation robotic systems have been equipped with a variety of sensors, which can measure eye motion, quality of hand-eye coordination, force and motion, grasp pressure profile, and neuromuscular activities such as electromyography (EMG) and electroencephalography (EEG).
b) A task-oriented visual game-like virtual reality environment, which is an inherent component designed to provide patients with multimodal cues during tasks, with the goal of enhancing the engagement and participation needed for promoting plasticity.
c) Programmable virtual therapist algorithms that are coded to provide intervention, and are responsible for quantifying the performance of the patients (based on the recorded multimodal data) and, accordingly, designing therapeutic reactions for delivery by the interface.

There are several advantages with the use of robotic technologies and they have shown potential in accelerating neural recovery. These technologies have been shown to enhance the quality of motor performance for stroke patients with mild-to-moderate disabilities. The contributing factors are as follows:

a) Power: Robots are powerful and precise, so they can generate accurate high- and low-intensity assistive and resistive force fields and vibrotactile haptic feedback to deliver therapy for a wide range of patients with various biomechanics over a long period of time.
b) Repeatability: Robots can be programmed to repeat an interactive task for as many iterations as are needed.
c) Objective assessment and progress tracking: Robots are computerized and can measure and log multimodal data, such as kinematic and kinesthetic factors (such as motion and force profiles in different joints), eye motion, quality of hand-eye coordination, biological signals (such as EMG and EEG); with the recording of all these modalities synced and saved for each session during rehabilitation. This enables precise and repeatable objective assessment that is imperative for clinicians to tune the dose, strategy, type, and intensity of therapy while monitoring the progress of motor enhancement.
d) Multimodal Stimulation for Engagement: Using VR environments coupled with robotic systems, visual, haptics, and auditory cues can be fused with kinesthetic rehabilitation, enabling multimodal goal-oriented sensorimotor tasks which can help to keep patients engaged and urge them to use their decision-making capabilities, which is a critical factor for stimulating neural recovery, in comparison to passive limb movement therapy.

Please see: Jimenez-Fabian and Verlinden. (2012); Chen et al. (2013); Tucker et al. (2015); Atashzar et al. (2019), for more details on these technologies. The effectiveness of robotic rehabilitation systems in enhancing neural recovery has been widely studied and attracted a great deal of interest in the literature (Krebs and Hogan. (2006); Atashzar et al. (2019); Bao et al. (2019); Simbaña et al. (2019); Shi et al. (2019)). There are several journals, societies, and conferences focusing on this topic to raise awareness regarding new robotic solutions, algorithms, technologies, and industries. However, despite the proven potential, there exist several challenges limiting the performance, efficacy, accessibility, compatibility, and usability of this technology. This has resulted in conflicting clinical studies with contradictory conclusions on the topic (Atashzar et al. (2019)). Based on the literature mentioned, among the limitations are 1) the restricted interpersonal interaction between the patient and the therapist, 2) a homogeneous response (with minimum flexibility) of a programmed robot over the workspace to a heterogeneous symptom space of the pathology, 3) non-standard strategies to tune the intensity, dose, and parameters of robotic therapy, 4) conservative constraints limiting the performance of the robot due to basic patient-robot safety features, 5) cost, accessibility and portability of robotic rehabilitation.

### 4.3 In-Home Robots for Delivering Rehabilitation During the COVID-19 Pandemic

Considering the current pandemic and the above-mentioned risks associated with visiting rehabilitation centers for post-stroke patients, while considering the imperative need for early rehabilitation, existing robotic systems can play a central role if their use is managed systematically. During the last decade, there has been an active scientific movement to make robotic systems home compatible (Huang et al. (2016); Bernocchi et al. (2018); Díaz et al. (2018); Washabaugh et al. (2018); Lyu et al. (2019)). For this, the three main factors to be met are safety, portability, and cost. Current commercial robotic rehabilitation systems are not primarily designed to be used in patient’s homes. Therefore, the existing commercial robotic rehabilitation systems are mostly expensive, bulky, and may not be safe enough to be used at home (with minimal supervision of an expert or trained operator). Safety is a major concern due to the ability of these technologies to generate very large forces while tightly connected to patients’ biomechanics (Zhang and Cheah. (2015); Atashzar et al. (2016b); Atashzar et al. (2017b); Atashzar et al. (2020)). In order to address these issues, two categories of suggestions have been made and implemented in the literature, 1) hardware solutions and 2) algorithmic solutions. Suggestions regarding hardware solutions have resulted in the design and implementation of novel robotic systems with inherent safety. In this regard, soft robots (please see Chu and Patterson. (2018); Cianchetti et al. (2018) and references therein) and mobile robots (see examples: Avizzano et al. (2011); Yurkewich et al. (2015); Germanotta et al. (2018)) are two suggestions in the literature, which be explained below. It should be noted that both soft rehabilitation robotic systems and mobile robotic systems can be made in very compact sizes at a low cost. One major reason for this is that both of these technologies drop the need for the use of heavy, expensive, motors in a rigid link format, which was previously required for delivering high-torque therapeutic forces.

a) Soft Robots: Soft robotic systems are composed of soft actuators, soft bodies, and possibly soft sensors. These robots are inherently safe due to their particular physics. Soft robotic systems are also usually inexpensive and can be made in small sizes, in particular in the format of soft exo-suits, which are soft exoskeleton robotic systems. These robotic systems can be operated with minimal concerns about safety (due to their compliant design) and can be used for a variety of rehabilitative tasks (Chu and Patterson (2018); Cianchetti et al. (2018)). These systems have great potential to be used in the homes of patients with NMSK disabilities, allowing them to have inexpensive rehabilitation therapy and minimizing the need for frequent visits to clinic.
b) Mobile Robots: Mobile wheeled robotic systems have been recently been considered as another potential solution to enhance safety and portability while reducing costs (Germanotta et al. (2018); Avizzano et al. (2011); Yurkewich et al. (2015)). The actuation principal of these robots is based on the friction between the wheels of a mobile platform and a table-top surface (instead of a robotic-links rigidly connected to a structure). Because these robots are not connected rigidly affixed to a base, they can provide a high degree of safety. In addition, since these systems do not require long arms and have indirect power transmission, they can be designed in a very compact size for maximum portability, while reducing the cost of the system.

In terms of algorithms, it should be noted that there has been active research on designing intelligent stabilizers (such as those designed based on the Strong Passivity Theory) which can guarantee the safety and stability of mechanisms by monitoring and updating the amount of energy which can be delivered and absorbed by patients’ biomechanics when conducting rehabilitation exercises (Zhang and Cheah. (2015); Atashzar et al. (2016b); Atashzar et al. (2017b); Atashzar et al. (2020)). These algorithms mainly function by monitoring the mechanical energy flow between patient and robot. By analyzing system stability conditions on the fly, these systems allow for initiation and tuning of interventions (through immediate injection of damping factors) whenever stability conditions are about to be violated. With the use of such intelligent observational algorithms, the safety and stability of HRI is guaranteed, adding one more layer of safety in addition to mechanical safety, as explained before. It can be envisioned that with the use of existing soft and mobile robotic systems, that have embedded intelligent stabilizers, we can have in-home robotic technologies to deliver a highly transpicuous kinesthetic therapy for patients in the home and minimize the need for visits and therapist-patient physical contacts. Considering the need for urgent rehabilitation post-stroke, and due to the extensive research and available mechanical and algorithmic supports, implementing such composite technologies on a large scale can be envisioned to address the lack of rehabilitation services for post-stroke patients in isolation due to the concerns related to COVID-19. Achieving this goal requires a focused interaction between industries, designing robotic systems, and healthcare systems, to make such technologies widely available for the public and maximizing the accessibility of rehabilitation services. This section provides the needed facts and scientific perspective of such discussion.

### 4.4 Telerobotic Rehabilitation: A Potential Transformative Paradigm for Delivering Supervised Remote Therapy

Telerobotic rehabilitation systems (under the category of teleoperated robotic systems) are the result of a natural extension of conventional robotic rehabilitation systems and have been seen as a novel paradigm within telemedicine, can maximize equal opportunity regardless of geographical constraints (Atashzar et al. (2016a); Shahbazi et al. (2016); Atashzar et al. (2018); Panesar et al. (2019); Hooshiar et al. (2019); Fong et al. (2020a); Fong et al. (2020b); Sharifi et al. (2020)) and restrictions caused by COVID-19. Telerobotic rehabilitation systems are composed of two synchronized robotic systems that communicate over a communication channel, (e.g., internet). One robot is at the patient’s side and one robot is at the therapist’s side. A virtual reality environment is shared between the therapist and the patient. As a result, the patient can perform tasks (like what he/she would do using conventional robotic systems), but at the same time, the motions are sent to the clinician’s side where the therapist can feel all the motions provided by the patients (since the two robots are synchronized in the position-force domain) and can react by applying forces. The forces generated by the therapist are logged using the sensory systems of robotic system while being sent back to the patient-side robot. The patient can move the robot, and the forces relayed to patient-side robot allow for the patient’s motion to be corrected and guided if needed. This technology can be a core solution for patients at home, since a remote therapist can interact with a patient not only through vision and audio channels (conventional telemedicine modalities) but also through kinesthetic and haptic interaction, which is imperative in the rehabilitation domain. With the use of this new paradigm, patients can benefit in-home from remote multimodal and tele-kinesthetic interaction with in-hospital therapists. This enables supervised and remote motor assessment and delivery of rehabilitation. This technology can realize the immersive experience of teletherapy and interpersonal interaction between the patient and the therapist. At the time of the COVID-19 crisis, the need for this technology is pronounced, which can significantly enhance the current state of telemedicine. Such technology enables wide-range interaction between clinicians and patients across the country with a specific focus on patients in nursing homes, those with co-morbidities, and those in areas with highly pressurized healthcare systems. This offers a transformation to equal access of healthcare services and is a major global need, especially during this crisis. Besides accessibility, telerobotic rehabilitation can significantly increase the duration in which a patient can receive rehabilitation services in-home since the involvement in a rehabilitation program would no longer be linked to physical visits to care centers.

It should be emphasized that although the concept of telerobotic rehabilitation has been proposed and investigated during the last decade, there were some restrictions, in the past, for realizing such technology at large scale, mainly due to the sensitivity of the quality of therapy to the quality of service (QoS) of communication networks. This includes issues related to reliability and resiliency of communication and security of data transfer. In this regard, latency, jitter, and packet loss not only deteriorate the fidelity of therapy rendered for the remote patient, but can also result in “non-passive coupling” between the two robots, adding to concerns about safety (as this can potentially cause asynchronous growing of interactional trajectories). This concern has been addressed in the literature to a reasonable extent, mainly 1) through the use of passivity stabilizers (mentioned earlier) and 2) accessibility to secure, highly reliable, and an agile internet connection, such as 5G and beyond Aijaz et al. (2016).

It should be noted it is imperative for therapists and clinicians to feel the kinesthetic actions and reactions of patients. This is needed for two major interconnected purposes 1) rehabilitation, 2) assessment, as explained below.

First, it should be mentioned that in the field of motor learning and rehabilitation sciences, it is known that a successful rehabilitative therapy needs to provide the therapist with the on-the-fly awareness of 1) the user-specific motor capability, kinematics, and biomechanical characteristics of the patient, 2) the specific characteristics of the neuromuscular deficits, and 3) the rate and pattern of motor improvement. These three factors are identified in the literature of rehabilitation as the three critical factors of motor retraining, which basically require physical interaction between therapists and patients. Thus it can be mentioned that although in-home autonomous robotic systems can deliver programmed rehabilitation therapy for patients in the home, without a telerobotic paradigm, these robots block the interpersonal interaction between a human therapist and the patients.

Second, it should be noted that interpersonal interaction is also known to be an imperative need, beyond rehabilitation, and specifically for long-term assessment of the severity of the condition and any changes in motor performance potentially correlated to the delivered regimen of rehabilitation.

Considering this note, the importance of telerobotic rehabilitation and assessment systems is further underscored. Thanks to the high speed, reliability, and accessibility of modern internet in many parts of the world, telerobotic rehabilitation can multiply the use potential of a therapist’s time by bypassing the obstacles due to distance and challenges due to isolation/quarantine situations caused by COVID-19. These technologies minimize actual human-human contact through virtualization, while still allowing computerized physical interaction. Considering the available communications backbone and robotic technologies, telerobotic rehabilitation can be envisioned as part of the response to the COVID-19 pandemic and to prepare healthcare systems for future pandemics. This section displayed the imperative need and feasibility of such telerobotic rehabilitation systems, with the hope of increasing public and scientific awareness on the topic.

Remark: It should be noted that one of the challenges which should be addressed for a fluent translation of telerobotic rehabilitation technology into practice is the cost and portability of robotic systems for use in the patient’s home (as one terminal of the telerobotic system). This is an active line of research and can be considered as the current limitation. However, due to the accelerated trend of improvement regarding in-expensive robotic systems, such as soft and mobile robotic technologies, which can be used in the context of rehabilitation to reduce the cost and improve the portability (as mentioned in the previous section), it can be envisioned that the mentioned limitations can be addressed in the near future. However, this would require further research, development, and investment in the future of telerobotic rehabilitation systems.

## 5 Assistive Technologies

As mentioned in the previous section, robotic systems have transformed the delivery of rehabilitation therapies, assisting with the gradual recovery of patients with sensorimotor disabilities. The other related, yet different, category of robotic systems developed to help patients with NMSK deficits are assistive robotic technologies. The primary difference is that assistive technologies are designed to immediately augment the sensorimotor capacity of NMSK patients and help them in performing activities of daily living. As a result, a gradual recovery is not the primary focus of assistive technologies. Assistive technologies are realized in various modalities of interaction, including smart wearable mechatronics (Simon et al. (2015); Chen et al. (2017); Lopes and Baudisch (2017); Maisto et al. (2017); Bisio et al. (2019); Yang et al. (2019a); Alva et al. (2020); Cerqueira et al. (2020); Gathmann et al. (2020)), handheld robots (Pathak et al. (2014); Stamford et al. (2015); Sabari et al. (2019)), exoskeletons (Gopura et al. (2016); Pazzaglia and Molinari (2016); Young and Ferris (2016); Hill et al. (2017); Settembre et al. (2020)), and smart wheelchairs (under autonomous robots) (Chow and Xu (2006); Parikh et al. (2007); Leaman and La. (2017)). Assistive technologies can be as simple as smart IoT-based fall protection devices (Saadeh et al. (2019)), smart gait-aid goggles for Parkinson’s patients (Ahn et al. (2017)) and active canes (Lachtar et al. (2019)); they can be also be more complex, such as exoskeletons (Gopura et al. (2016); Pazzaglia and Molinari (2016); Young and Ferris (2016); Hill et al. (2017); Settembre et al. (2020)). In this regard, it should be noted that falls are a major concern for the aged population (Terroba-Chambi et al. (2019); Silva de Lima et al. (2020)) and can result in critical bone fractures (which heal slowly, if at all) and other deteriorating secondary conditions. On the other hand, mobility is essential for aged individuals to maintain cardiovascular and musculoskeletal health, particularly after recovery from NMSK conditions. This is an addition to the normal needs for situational awareness and navigation in daily living environments and manipulation of objects (such as doorknobs, food, etc.). Addressing this need to enable mobility without the use of advanced technologies would call for more interaction with care providers for the delivery of assistance, which increases the risk of infection transmission among this vulnerable population. The main outcome of the use of assistive systems is enhanced situational awareness (i.e., perceptual augmentation), enhanced independence, empowered mobility, and increased manipulability for individuals with degraded sensorimotor competence, (i.e. motor augmentation).

Common use cases of assistive robots to improve the motor performance of patients living with NMSK are 1) exoskeletons for patients with spinal cord injuries, stroke, and gait deficits, 2) smart motorized wheelchairs for patients with severe lack of mobility, 3) wheelchair-mounted arms for patients with the lack of manipulability (such as those aging with severe cerebral palsy), 4) smart motorized walking supports for patients with limited mobility and those with a high risk of fall, and 5) handheld tremor compensators for patients with pathological hand tremors such as Parkinson’s disease and essential tremor.

In addition to the above-mentioned examples, which mainly focused on augmenting the motor performance of users, the second category of assistive mechatronic technologies are designed to augment the sensory perception of the patients. These active smart-technologies aim to boost up the perceptual awareness of users, to improve perception of sensory input. These technologies ultimately help with activities of daily living and tracking the health status of patients. Sensory perception enhancing systems may be in the format of wearable suits, (e.g. armbands) and may provide auditory, vibrotactile, or visual cues for the patients. One example of such a systems are wearable vibrotactile suits for helping individuals with degraded vision and sensory awareness, so they can navigate safely in daily environments while protecting them when encountering unexpected contacts, which may result in falls (Bharadwaj et al. (2019)). Another example is technologies that provide cues to the user regarding their posture during walking to maintain a safer balance (Viseux et al. (2019)). These technologies have been used to enhance sensory awareness of people with degraded vision and perceptual capability. Another important example is closed loop and open loop sensory cueing systems for patients with freezing of gait caused by Parkinson’s disease (Mancini et al. (2018); Sweeney et al. (2019)). Freezing of gait can result in danger and major challenges during daily navigation (such as crossing a street, navigating in a home, walking to the bathroom, etc.), resulting in limited mobility and independence. With the use of sensory augmentation technologies, patients with Parkinson’s disease have shown to have significantly enhanced mobility and have recovered a high degree of gait fluency. This is believed to be caused through the opening of a redundant neural sensory processing pathway, which may be less affected by degenerated neurons. The above-mentioned technologies will enhance the mobility and independence of patients with NMSK conditions, minimizing reliance on caregivers, which reduces concerns of disease transfer. Additionally, new assistive and wearable technologies have been recently proposed to increase gesture awareness to alert individuals about hand-face contact to reduce the risk of COVID-19 infection (D’Aurizio et al. (2020)). Although some of these technologies may not be directly categorized as robotic systems, they are smart mechatronic modules that can enhance sensorimotor functionality of people, while minimizing the risk of infection and maximizing the patient’s cognitive awareness about the possible risky situations (which should be strictly avoided for NMSK patients with co-morbidity).

Enhancing motor performance and situational awareness, offered by assistive technologies, is particularly critical during the COVID-19 pandemic, as the increasing a person’s independence during daily activities decreases their need for interaction with helpers, nurses, and care providers. In other words, using assistive technologies, patients with sensorimotor deficits require a lower amount of supervision and physical interaction with care providers for conducting activities of daily living. This can also reduce the need for having a high number of nurses and helpers in long term care facilities, which is a significant concern at the moment with concerns related to bilateral disease transfer between patients and between patients and care providers. Besides cognitive aspects, there are several mobility/manipulability restrictions that are associated with normal aging or age-related NMSK deficits. This includes gait control problems, balance problems, dexterity deficits, lack of motor power, affected precision in targeting, perceptual deficits, and involuntary movements.

Thanks to the use of advanced assistive technologies, the need for interpersonal interaction between elderly and care givers can be significantly reduced. This shows an unmet need to boost the performance, and availability, of assistive technologies to help patients with conducting many activities of daily living. With the use of advanced smart assistive robotic and mechatronic technologies, it is possible to enhance mobility and manipulability during the daily lives of senior individuals; ultimately improving their independence and increasing their situational awareness while minimizing the risk of COVID-19 infection. By employing several assistive technologies, the need for care providers in the living environment of senior individuals will be reduced, minimizing the risk of infection transmission to this vulnerable population during and after the COVID-19 pandemic era. Due to the strong literature and successful implementation of assistive technologies, short and long-term investment in this field of research and development can make the healthcare system more prepared for future pandemics.

## 6 Robots for Assessment and Support

In this section, we discuss the use of robotic and mechatronic technologies for 1) delivering assessment for monitoring, evaluating, and diagnosing NMSK disabilities and 2) for providing mental, social, cognitive, and emotional support to isolated NMSK individuals. Support and assessment technologies can be implemented in a number of ways through robotic and wearable technologies. These technologies are grouped together here as many supportive technologies require some manner of real-time monitoring or assessment of an individual.

### 6.1 Social Robots for Support

It should be noted that due to COVID-19-related guidelines and concerns, the elderly, particularly those with age-related NMSK disabilities and mobility issues, are affected by extra social distancing and prolonged isolation policies. This leads to secondary challenges such as depression, anxiety, and stress, caused by excessive and prolonged isolation in this population (Armitage and Nellums. (2020)). Seniors are being isolated from their families and caregivers, with some long term facilities around the world reducing or restricting patient/physician visits. Given this, robotic and wearable technologies can be used to compensate in part for this lack of direct physician, caregiver, and family interaction. Social robots, for instance, are designed to interact and communicate with humans and their surrounding environment. Social robots have been constructed in a range of form factors from pet-like toys (e.g., Paro) to humanoids (e.g., Sophia). Social robots have been shown to be particularly effective at helping with the mental health and well-being of elderly persons with dementia or other NMSK conditions in healthcare and long-term care settings (see Pu et al. (2019); Scoglio et al. (2019)). Social robots can provide or act as a companion to help people with NMSK conditions feel less lonely, feel more socially engaged, and interactive. Social robotics has primarily been used in assisting with the treatment of elderly patients, particularly those with dementia, and have been shown to have a positive benefit in improving mood, reducing anxiety, and reducing depression.

The mood-boosting effects of social robotics can be particularly helpful during the COVID-19 pandemic, as social robots can help to bring a sense of comfort and interaction to isolated elderly persons, and can be used to create a sense or routine or order without the need for caregiver interaction. From its inception, social robotics research traditionally has been focused on robotics for elderly care and those with NMSK disabilities. Social robots have gained new relevance during the pandemic, with many seniors, group, and long-term care homes no longer allowing family members (or with extreme restricted care and reduced frequency and physical contact), social workers, and support workers to visit. Due to the low-cost and substantial research that has already been done with social robotics, they are among the technologies that can be quickly deployed to healthcare and long-term care settings during the COVID-19.

### 6.2 Mechatronic Assessment Technologies

Smart wearable mechatronic technologies refer to smart body-worn devices that can measure, analyze, display, and transmit information and are among other smart mechatronic technologies which can significantly reduce the burden on the healthcare system. Due to the close physical contact with the body, these devices have been used to measure several biomarkers of users, including heart rate, oxygen saturation level, temperature, and mobility. Monitoring these biomarkers is imperative for remotely supervising the health status of isolated seniors and, in particular, those in long term care facilities. These technologies can help to find, diagnose, track, and trace COVID-19 symptoms and infections. They can directly assist the healthcare system to more optimally distribute resources and act quickly to 1) avoid the worsening of the symptoms, 2) avoid transmission of COVID-19 among elderly adults, especially in long care facilities. Due to the computational power available to modern cloud processing modules, data collected using wearables can be processed on the fly with machine learning systems. Thus, such technologies have been suggested for detecting and tracking COVID-19 symptoms and alerting of any anomalies (Seshadri et al. (2020)). They have also been used for contact tracing and activity tracking of patients during the COVID-19 pandemic to monitor adherence to guidelines for protecting individuals and reducing the spread of infection (Pépin et al. (2020); Seshadri et al. (2020)).

Besides being used for monitoring and assessment of health status and searching for COVID-19 symptoms/infections, such technologies can be used to remotely monitor the physical performance of patients with NMSK conditions (Venkataraman et al. (2017); Noorian et al. (2018); Noorian et al. (2019); Sanders et al. (2020); Venkataraman et al. (2020)). Using such technologies, the need for frequent visits to clinics for (subjective) recording of patient performance would be minimized, further reducing the risk of disease transfer during the pandemic. A classic example of these devices is those that monitor (and encourage) physical activity (for instance a Fitbit watch). More complicated wearable devices can monitor patients physiotherapy exercises in-home as part of telemedicine services. They may also monitor vital signs, or report if a person is in distress through the detection of serious conditions such as fall(s) and monitoring of mobility status. For elderly people with NMSK conditions, there is a clear benefit to using wearable technologies to keep track of rehabilitation progress and quality of life measures without requiring hands-on contact with a clinician or rehabilitation specialist. Many of the interfacing sensors (such as EMG, MMG, and EEG) can be built into wearable devices opening an unobtrusive neurophysiological window to the underlying biomarkers. Thus allowing for a truly remote and objective assessment of patients with NMSK conditions in their homes, while relaxing the need for in-person visits (please see Maceira-Elvira et al. (2019) and references therein). This is a critical factor to be considered that can allow the clinician to monitor the progress of and recovery after a NMSK condition, such as stroke.

Research in both fields of social robotics and smart wearable monitoring mechatronics have had significant progress during the last decade resulting in a wide range of available, inexpensive, technologies which can be exploited by the healthcare system in the short-term future to further support patients. Particularly those in need of NMSK rehabilitation, supervision, and monitoring. Thus with systematic planning and involvement of stakeholders, such technologies can be utilized to fight the primary and secondary challenges imposed by the COVID-19 pandemic for serving patients with underlying NMSK conditions. The proven potential for such technologies calls for further investigation and development to provide a range of “standardized” devices to lift the pressure on healthcare systems in future potential waves of the COVID-19 pandemic and potential future pandemics.

## 7 Concluding Remarks

The COVID-19 pandemic has significantly affected the healthcare systems and has raised several questions about its capacity and preparedness to serve under heavy pressure. Based on the significant advancements in various fields of engineering, it is widely accepted that the current unprecedented pressure could have been eased if available technologies, developed during decades of research and investment, had been channeled through a standardized pipeline to tackle the many challenges presented by existing conditions before the pandemic. Among these challenges, there is a growing concern regarding services needed for patients with NMSK conditions, many of which are halted, whilst treatment is still extremely time-sensitive (such as rehabilitation post stroke). In this perspective review article, we have provided a detailed analysis of existing technologies and literature, and discussed the corresponding capacity and how they can help to serve patients, particularly those in the three critical domains of NMSK care (namely rehabilitation, assessment, and assistance). Supported by current literature, we believe that there exists significant technological advancements that could have been established and deployed to deliver a much higher quality of care for NMSK patients during the COVID-19 pandemic. We have provided a detailed discussion of several examples of such technologies and introduced their capacity. This article provides an in-depth and focused look at the existing literature and provides a platform, and the needed information, to initiate a conversation between stakeholders, engineers, policy makers, researchers, and healthcare providers to discuss various aspects of intelligent robotics and smart mechatronic technologies to augment the delivery of care through a systematic investigation, investment, and development for NMSK patients. We believe that the existing technologies have the ability, and are ready, to assist with healthcare delivery during the current and upcoming future waves of the pandemic, if much needed awareness is raised. In addition, this article strongly suggests that a continual conversation be struck, so that for future pandemics, healthcare systems can be equipped with the power and intelligence of robotics and mechatronics technologies to ensure patients with NMSK conditions receive the same high level of care comparable with the that received during the pre-pandemic era.
